# Seasonality and the persistence and invasion of measles

**DOI:** 10.1098/rspb.2006.0030

**Published:** 2007-02-27

**Authors:** Andrew J.K Conlan, Bryan T Grenfell

**Affiliations:** 1 DAMTP, Centre for Mathematical SciencesWilberforce Road, Cambridge CB3 0WA, UK; 2 Center for Infectious Disease Dynamics, The Pennsylvania State University208 Mueller Lab, University Park, PA 16802, USA; 3 Fogarty International Center, US National Institutes of HealthBethesda, MD 20892, USA

**Keywords:** measles, persistence, critical community size, birth rate, invasion, vaccination

## Abstract

The critical community size (CCS) for measles, which separates persistent from extinction-prone populations, is arguably the best understood stochastic threshold in ecology. Using simple models, we explore a relatively neglected relationship of how the CCS scales with birth rate. A predominantly positive relationship of persistence with birth rate is complicated by the accompanying dynamical transitions of the underlying deterministic process. We show that these transitions imply a lower CCS for high birth rate less developed countries and contrary to the experience in lower birth rate, industrial countries, the CCS may increase after vaccination. We also consider the evolutionary implications of the CCS for the origin of measles; this analysis explores how the deterministic and stochastic thresholds for invasion and persistence set limits on the mechanism by which this highly infectious pathogen could have successfully colonized its human host.

## 1. Introduction

A key process in population biology is the local extinction of small populations arising from chance. The inherent unpredictability of the timing of basic demographic processes such as birth, death and migration leads to frequent extinctions in populations below a certain threshold size. This threshold, which separates persistent populations from those vulnerable to such *demographic stochasticity*, is characterized empirically for only very few systems (Hanski & Gaggiotti 2004). Arguably, the best documented and understood is the critical community size (CCS), which defines the *stochastic* persistence of measles as a function of human population size (Bartlett 1957; Black 1966).

Effective vaccination campaigns have reduced measles to low levels in many industrial countries (Wallinga *et al*. 2001). On the contrary, the disease is still a killer in high birth rate less developed countries, and monitoring and reporting must necessarily take second place to more urgent public health issues. Theoretical advances have been made in estimating deterministic eradication thresholds (McLean & Anderson 1987*a*) and the impact of vaccination for measles in less developed countries (McLean & Anderson 1987*b*). Stochastic persistence, however, has not been examined systematically for high birth rate communities. Insights from industrial countries are limited, as the turnover rate of developing country is much larger—the birth rate in Sub-Saharan Africa can be up to three times larger than in the UK (US Census Bureau). This leads to typically annual dynamics before vaccination in contrast to the usually biennial dynamics observed in industrial countries (McLean & Anderson 1987*a*; Broutin *et al*. 2005).

In this paper, we use simple models to explore how stochastic persistence scales with birth rate, and therefore the recruitment rate of susceptibles. We show powerful effects of birth rate on persistence; however, seasonality and dynamics modify this picture, with potentially significant consequences for vaccination campaigns. This exploration is also relevant to the persistence of morbillivirus across animal populations with different demographies (Swinton *et al*. 1998).

The interplay between the measles virus and its host is among the best understood population dynamic interactions (Anderson & May 1991), in part due to the infection's simple life history (Black 1984). A viral infection that is spread by aerosol transmission leads to a characteristic red rash appearing on the body approximately 8 days after infection. The onset of clinical symptoms marks the beginning of the infectious period, which lasts for approximately 5 days. Although various genetic strains of measles are known to exist, recovery from infection from one strain leads to a lifetime immunity to reinfection from all others (Woeld *et al*. 2001). This combination of long-lasting immunity and a high infection rate results in measles being a predominantly childhood disease and intimately connects the infection dynamics and the birth process.

The short time-scale of this progression to immunity leads to relatively violent epidemic cycles, followed by deep troughs in measles abundance. In essence a simple oscillator, the dynamics are strongly influenced by seasonal variation (forcing) of the transmission rate. In the best documented case of the UK, forcing depends on the seasonal aggregation of children in primary schools (Fine & Clarkson 1982*a*); thus, the pattern of mixing with age is also potentially important (Schenzle 1984), though much can still be learned from simple non-age-structured models (Earn *et al*. 2000; Bauch & Earn 2003). In the context of many less developed countries, more general seasonal drivers than schooling, such as the agricultural cycle and associated droughts and famines, are likely to play a more significant role (Duncan *et al*. 1997).

The development of this dynamic understanding for industrialized countries has benefited from rich databases of measles incidence from the UK, the USA and elsewhere (Cliff *et al*. 1993; Grenfell *et al*. 1995; Ferguson *et al*. 1996). The resulting theoretical models have been used to aid policy and predict the efficacy of vaccination campaigns (McLean & Anderson 1987*b*; Babad *et al*. 1994; Edmunds *et al*. 2000; Wallinga *et al*. 2001). The combination of data and models also allowed Bartlett, Black and others to characterize a CCS of 250–500 000 for urban communities (Bartlett 1957; Black 1966; Keeling & Grenfell 1997). The epidemic dynamics and seasonal forcing, spatial heterogeneity (Bolker & Grenfell 1995; Lloyd & May 1995; Earn *et al*. 1998; Grenfell *et al*. 2001; Xia *et al*. 2004) and the details of the infection and recovery process (Keeling & Grenfell 1997, 2002; Lloyd 2001*b*) have been shown to be important in understanding the level of the CCS.

### (a) Thresholds for persistence

A clear distinction should be made here between the CCS (the stochastic threshold for persistence) and the deterministic thresholds for invasion and eradication.

In a deterministic epidemic model, a disease can only invade a fully susceptible population if the basic reproductive ratio *R*_0_—defined as the average number of new infections per infective (Anderson & May 1991)—is greater than unity. As an epidemic progresses, the accumulation of immune individuals in a population, which drives the so-called herd immunity, reduces both the proportion of the susceptible (*s*) population and the number of new infections generated by each infective to give an effective reproductive ratio *R*=*sR*_0_. The aim of vaccination campaigns is to reduce the level of susceptibility in the population, such that *R* becomes, and remains, less than unity. This is known as the *eradication* or *invasion* threshold, since the infection not only becomes extinct but can also no longer re-invade from an external source.

However, for small isolated populations within a larger community where measles is endemic, demographic stochasticity can lead to the extinction of the pathogen in the deep troughs between epidemics even when *R* is greater than unity. Given the seasonal fluctuations in abundance of measles, the appropriate threshold for the *stochastic* persistence of measles is therefore a *host* population size.

### (b) Thresholds for measles invasion

A much-cited conceptual use of the CCS is in providing insights into the necessary condition for the measles virus to have invaded human populations. The most probable origin is an escape mutant of the cattle disease rinderpest or canine distemper (Westover & Hughes 2001). Black was the first to point out the possible evolutionary implications of the CCS, suggesting that it also provided a threshold for the original successful invasion of measles into the human population (Black 1966). Working on this assumption, and supported by the apparent evolutionary stability of measles and the absence of animal reservoirs, he suggested that the human population would first have exceeded this threshold during the rise in the ancient Mesopotamian civilizations 2000–4000 years BC. The link between crowd diseases such as measles and the rise in urbanization has already been discussed at length (Cliff *et al*. 1993; Diamond 1997, 2002). Pre-demographic transition societies would have had a demography much more akin to less developed countries today with a correspondingly high turnover population (McCormack Adams 1981). We therefore use our less developed country analysis to explore how the stochastic threshold of high turnover populations may have influenced measles invasion.

However, the concept of the CCS applies to measles dynamics about an endemic equilibrium. First, the invading disease has to survive a deep trough following its ‘virgin soil’ epidemic in the wholly susceptible human population (Anderson & May 1991). For a disease to invade and persist to become *endemic*, the susceptible proportion *s* remaining after the first epidemic must be large enough, such that *R* is still greater than unity. We conclude the paper by showing how the successful initial survival of an infection such as measles depends on a combination of the deterministic invasion and stochastic persistence thresholds.

### (c) Model

To explore the relative change in persistence with birth rate, we use the simplest possible standard compartmental model with constant population size and equal birth and death rates. Individuals are labelled only by their epidemiological status—*S*usceptible, *E*xposed, *I*nfectious and *R*ecovered (SEIR; Anderson & May 1991). The model is defined by the rates of transition between these states, updated by continuous time Monte-Carlo simulation methods. The latent and infectious periods are assumed to be fixed at 8 and 5 days, respectively. The dynamics of the model are thus determined completely by the birth rate, *B*, into the susceptible class and the transmission rate, *β*(*t*), which is assumed to have a fixed annual variation of amplitude *α*.

The fundamental assumptions are as follows.

- Frequency-dependent mass action mixing (De Jong *et al*. 1995), which implies no deterministic threshold in the population size (*N*).
- Homogenous transmission rates with respect to age (Hamer 1906; Ross 1916; Soper 1929).
- Discrete (gamma-distributed) infectious periods using the method of stages (Anderson & Watson 1980; Keeling & Grenfell 1997, 2002; Lloyd 2001*a*).

Under these assumptions, *R*_0_ has a particularly straightforward form independent of population size, *R*_0_≈〈*β*〉/*r*: a function of the infectious period 1/*γ* and the time-averaged transmission rate, which we interpret as an effective transmission rate for a population. A simple transformation of the model equations reveals a scaling relationship, such that changes in birth rate *B*→*B*′ produce the same dynamical transitions as a proportionate increase in transmission rates (Earn *et al*. 2000)

(2.1) $$\beta \to \frac{B^{\prime }}{B}\beta .$$

Therefore, epidemic dynamics will remain invariant under a change in birth rate if the transmission rate is scaled proportionately; a result we will return to later. Given the well-known heterogeneities in transmission rates with age (Anderson & May 1991), this scaling relationship will only be valid for a limited range of birth rates. This and the other complexities introduced by age structure are discussed in detail in the electronic supplementary material.

Space is treated implicitly—we consider populations as locally isolated with infrequent imports of infection from an external source. A constant (small) stream of infectives into the population restarts epidemics after the fade-out. The exact quantitative pattern of fade-outs (and critically fade-out duration) depends on the number of immigrants and the chosen scaling with population size. We use Keeling's formulation, scaling with the square root of population size to maintain continuity with previous studies (Keeling & Grenfell 1997, 2002). Simulations with other formulations (Xia *et al*. 2004) generate the same qualitative results presented here.

## 2. Results

### (a) Changing patterns of persistence with birth rate

In developed countries, the deep troughs between major epidemics frequently lead to local extinctions, or ‘fade-outs’, of measles in communities below the CCS. The probability of extinction at any time is highly dependent on the past dynamical history of the system, so a natural measure of persistence is the mean annual number and length of fade-outs (Keeling & Grenfell 2002). We employ Bartlett's definition of fade-out of three or more weeks with no new reported cases (Bartlett 1957). In figure 1*a* we use the stochastic SEIR model to plot theoretical curves for mean annual fade-outs against population size for the UK birth rate alongside equivalent curves for representative higher birth rates.

Despite the simplicity of the model, a birth rate of 20 per thousand captures the qualitative pattern of annual fade-outs in England and Wales (black in figure 1*a*) and their relationship with population size (although slightly overestimating the CCS and the number of fade-outs). Age-structured models have provided closer matches for the CCS for England and Wales (Keeling & Grenfell 1997), but the added model complexity only obscures the simple mechanism explored here: we are interested in the *relative* scaling of the CCS with birth rate, which is unaffected by the use of the simple model (for a further discussion see the electronic supplementary material).

Intuitively, we might expect that raising the birth rate would correspondingly increase persistence, elevating the depth of the troughs between epidemics and reducing the probability of fade-out. Indeed, doubling the birth rate to 40 per thousand dramatically reduces the CCS (green in figure 1*a*); however, at an intermediate birth rate of 30 per thousand, the CCS actually *increases* (red in figure 1*a*). As shown in figure 1*b*–*d* these changes in persistence result from dynamical transitions between three main regimes of periodicity: annual (high birth rate, figure 2*c*); biennial (intermediate birth rate, figure 2*b*); and multi-annual (low birth rate, figure 2*a*).

Since we allow the measles population to become extinct, we need a more general definition of trough and peak size than the maximum/minimum values of a replicate. We define a distribution of expected weekly cases (see electronic supplementary material) from which we use the upper and lower tails to characterize average trough and peak sizes for a given birth rate (figure 2*d*–*f*). Annual epidemics result in much higher troughs, greatly reducing the impact of demographic stochasticity. Under the assumption of mass action mixing, the underlying deterministic dynamics scale with population size. The change in trough size for a large population, above the CCS, will therefore be an index for the relative changes in persistence imposed by the change in periodicity.

To explore this change in persistence more systematically, we consider how the pattern of fade-outs changes over a range of birth rates for human communities. The mean numbers of annual fade-outs were calculated from model simulations for a range of population sizes from 10 000 to 3 million and plotted against birth rate in figure 1*b*.

This plot confirms our expectation of a positive relationship between the persistence and the recruitment rate, except for the ‘bump’ of lowered persistence for birth rates between 15 and 30 per thousand; counter-intuitively, the CCS doubles with birth rate over this range. We can quantify the relative mixture of annual and multi-annual cycles identified in figure 2 in terms of the average power of these modes in the Fourier spectrum (figure 1*c*). At low and high birth rates, we see the expected dominance of biennial and annual dynamics, respectively. The high CCS at intermediate birth rates corresponds to the high amplitude biennial dynamics between these regimes. Plotting the trough and peak size of the largest population size above the CCS (figure 2*d*) shows that the associated minimum in the trough size of incidence corresponds to a maximum in the peak incidence. Increasing birth rates effectively ‘pumps’ the major epidemic of the biennial cycle to a greater peak, resulting in correspondingly lower incidence in the following troughs. The effect of the increased recruitment of susceptibles is offset by the redistribution of cases within the biennium. The critical birth rate, where the CCS is reduced dramatically, is caused by the well-known transition in the deterministic model to annual periodicity at a birth rate of around 30 per thousand, corresponding to much higher troughs between epidemics (Aron & Schwartz 1984).

To summarize, we see an overall positive relationship between the persistence and the birth rate. However, there is a significant reduction in persistence at intermediate birth rates. This stems from the deterministic transitions in periodicity and resultant change in the depth of troughs between major epidemics.

### (b) Vaccination: implications for persistence

Vaccination is a reduction in the recruitment of susceptibles and therefore essentially equivalent to reducing the birth rate (Aron 1990). It is a well-known theoretical result that changes in susceptible recruitment lead to commensurate and rapid changes in the epidemic period (Anderson & May 1991; McLean & Anderson 1987*b*). This effect has been borne out by the lengthening of the epidemic period during many reported vaccination campaigns (Agur *et al*. 1993; Earn *et al*. 2000; Santos *et al*. 2004). However, the impact of vaccination on the stochastic persistence of measles has been less studied. In England and Wales (low birth rate, high *R*_0_) moderate vaccine uptakes did not significantly alter the level of the CCS (Fine & Clarkson 1982*b*). From figure 1*b*, we see that for a high birth rate (high *R*_0_) population, a transition to biennial dynamics due to imperfect vaccination would result in a significant increase in the CCS.

The possibility of such transitions depends critically on the birth and transmission rates before vaccination. We can use the dynamic equivalence between birth and transmission rates (Earn *et al*. 2000) to explore the possible vaccine-induced dynamical transitions from a high birth rate country experiencing annual epidemics before the onset of vaccination.

The periodicity of measles dynamics in the SEIR model is determined by two key parameters: the effective transmission rate between susceptible and infectious pairs, *β*, and the amplitude of the seasonal variation in this rate, *α*. We can explore the possible persistence outcomes of vaccination by looking at the key statistics of periodicity and trough size for a plausible range of these parameters. In order to separate out only the impact of periodicity on trough size, we maintain a large population size at a fixed birth rate. Varying birth rates directly, as well as being computationally more intensive, will lead to a linear increase in the trough size, complicating the overall picture. The equivalence between changes in birth and transmission rates implies that this one diagram can illustrate the gross changes in the trough size for a realistic range of birth and transmission rates (figure 3*a*), which result from changes in periodicity (figure 3*b*).

At a fixed birth rate, high transmission rates will generate the same dynamics as in a population with a higher birth rate but lower transmission rates. For a given reference value of *R*_0_, we can rescale the *y*-axis of figure 3 in terms of a range of birth rates that will have equivalent dynamics. The scale for *R*_0_=20 is given; arbitrary values of *R*_0_ result in a direct rescaling of this axis. The expected change in the trough size with birth rate following the introduction of vaccination in England and Wales is consistent with the observation that the CCS was unchanged (Fine & Clarkson 1982*b*). Vaccinating 70% of births leads to a transition between biennial and multi-annual seasonality—the trough size, however, is of a similar magnitude to that before vaccination. The spatial decorrelation of epidemics (Cliff *et al*. 1992; Bolker & Grenfell 1996; Rohani *et al*. 1999) after vaccination (not considered here) was also a significant factor in this constancy of persistence. For other transmission parameters however, vaccination could lead to dynamical transitions, which would have a greater impact on the CCS.

A population will experience annual dynamics at high or intermediate birth/transmission rates (figure 3*b*). If we consider a high birth rate country with *B*=40 per thousand experiencing annual epidemics, the topology of this figure implies that three alternative scenarios can occur when vaccinating. (i) With low transmission rates (*R*_0_≈12) and small seasonal variation, there is a biennial phase but with a small amplitude; there are thus no associated dramatic changes in the trough size or persistence. (ii) With very low transmission rates (*R*_0_≈6) and moderate to high seasonal variation, there is a small island of annual seasonality between the multi-annual and biennial regions of parameter space. Vaccination from here would result in epidemics becoming more irregular and multi-annual, with correspondingly deeper troughs between epidemics (annual to multi-annual). The inclusion of a small degree of seasonal variation in transmission rates would change the dynamics sufficiently to increase the CCS. (iii) Finally, the most extreme case of high transmission rates (*R*_0_≈20) and high seasonality—essentially the UK parameterization discussed in this paper—could lead to a dramatic switch into biennial epidemics (annual through biennial).

In conclusion, to predict the dynamical impact of vaccination on persistence in a high birth rate country, it is essential to estimate the seasonal variation of transmission, as well as the more commonly calculated mean value. Although the CCS will always be increased by vaccination, the magnitude of this increase depends critically on the change in periodicity in incidence.

### (c) Persistence during invasion

Figure 2 shows that high birth rates (greater than or equal to 40 per thousand) could decrease the CCS for measles by a factor of 2. Given the high birth rates expected in pre-industrial societies, a much smaller human population than previously thought would have been sufficient for measles to have successfully persisted in early cities. However, these results relate only to the endemic steady-state after invasion has occurred. A large, dense, reasonably well-mixed population greater than the CCS is a requirement for measles to persist endemically. We show below that satisfying this *stochastic* threshold is in some ways mutually exclusive with the *deterministic* requirements for successful invasion.

In the few documented cases of virgin soil invasions of measles, Greenland in 1951 (Christensen *et al*. 1952, 1953, 1954), Fiji in 1875 (Squire 1875) and the Faeroe Isles in 1846 (Panum 1940), the rate of spread was so great that the entire population was infected within a matter of months. It is a well-known theoretical result from the simple epidemic model that for *R*_0_ values not much greater than 1, the majority of the susceptibles in the population will be infected in the first epidemic (Anderson & May 1991). Assuming the same natural history of infection as in the modern era, the short infectious and latent period of measles would imply high transmission rates and lead to a short first epidemic of the order of half a year. At the end of this epidemic, the susceptible population would be well below the deterministic eradication threshold.

The success of a virgin soil invasion of measles then depends critically on two key time-scales: the length of the first epidemic (essentially, the time to the first extinction of measles *T*_*E*_) and the time for the susceptible population to replenish through births to the epidemic threshold *R*>1(*T*_*I*_). We use a simple deterministic model to explore the lower bounds on these time-scales, neglecting the effects of mortality and stochasticity.

The time to first extinction (*T*_*E*_) is essentially determined by the length of the infectious and latent periods, with very little variation in transmission rate or birth rate (figure 4*b*). Raising *R*_0_ from 2 to 20 only results in shortening this epidemic by the order of 100 days. This is in sharp contrast to the time for the first possible re-invasion (*T*_*I*_ the first time that *R*>1), where there is a difference of 78 years between the lower and upper bounds of *R*_0_ considered (figure 4*c*). Unlike *T*_*E*_, the birth and transmission rates have a proportionate effect on determining *T*_*I*_, since the level of the invasion threshold is determined by *R*_0_; a higher *R*_0_ implying a lower invasion threshold and therefore shorter *T*_*I*_.

In summary, for well-mixed populations, self-immunizing diseases such as measles essentially eradicate themselves on the first invasion to a well-mixed population. Colonization and the transition to endemicity depends on a reintroduction to the population within the time-scales imposed by the recruitment rate and the deterministic invasion threshold. High birth and transmission rates, necessary for persistence in the endemic phase, are also beneficial during the transition from invasion.

## 3. Discussion

### (a) Persistence in less developed countries

We have shown that birth rate has a predominantly positive relationship with the CCS. This relationship is strong: a doubling of the birth rate leads to a fivefold reduction in the CCS. For small population sizes, increased birth rate always leads to greater persistence (figure 1*a*). The associated dynamical changes that birth rate has on incidence can lead to an enhancement or negation of this effect for populations close in magnitude to the CCS.

In the design and assessment of vaccination programmes, epidemiologists rightly concentrate on estimating the long-term average of the reproductive ratio *R* and the basic reproductive ratio *R*_0_. In this paper, we have demonstrated that seasonal variation in transmission can also have profound implications for the dynamics and hence stochastic persistence of childhood diseases. Dynamical transitions in periodicity of childhood diseases associated with changes in rates of recruitment have been extensively studied (Aron 1990; Earn *et al*. 2000; Grenfell *et al*. 2002), as have the association between the periodicity of outbreaks and persistence (Bartlett 1960; Yorke & London 1973; Bolker & Grenfell 1995). This study synthesizes these two deterministic and stochastic processes in a systematic fashion, with particular emphasis on applications to high birth rate countries.

In some populations with high transmission and birth rates, the lengthening of epidemic periods after vaccination could significantly raise the CCS in high birth rate countries, with significant consequences for the spatial spread of measles between isolated settlements. The travelling waves of measles observed in England and Wales (Cliff *et al*. 1993; Grenfell *et al*. 2001) were the result of infective ‘sparks’ from persistent urban hubs (above the CCS) restarting epidemics in smaller settlements after epidemic fade-out. With a much smaller CCS, we would not expect to see such complex dynamics in high birth rate countries before vaccination—in much the same way that the epidemiologically similar, but more persistent, whooping cough showed no evidence of spatial correlation before vaccination in England and Wales (Rohani *et al*. 1999). Such dynamics would become possible if vaccination raised the CCS sufficiently—large-scale changes in the global spatial dynamics of disease, which could not be predicted purely from the transmission rates before vaccination. Such transitions could help or hinder eradication campaigns (Earn *et al*. 1998), but cannot be predicted without directly estimating the amplitude of seasonal variation in transmission (*α*).

Estimation of *α* requires long-term, high resolution incidence data, very few examples of which exist for less developed countries. A recent study on measles and pertussis dynamics in Senegal is the best available example (Broutin *et al*. 2005). Focusing on metapopulation dynamics and persistence in small townships, they unfortunately did not have a large enough range of population sizes to estimate the CCS. Their estimate of *R*_0_≈4 before vaccination is well below the critical value for biennial epidemics, no matter what the value of *α* is—indeed, the onset of vaccination did not result in the dramatic changes in persistence predicted for the England and Wales parameterization explored here.

Overall, our study highlights the importance of gathering more detailed longitudinal incidence data in less developed countries (Broutin *et al*. 2005; Grais *et al*. 2006).

### (b) The origins of measles

Although our simple model framework can explain the dynamics of incidence and extinction well in the endemic regime, its application to the question of measles invasion raises a paradox as to how such an infectious disease could have successfully invaded and persisted. Although a reasonably well-mixed, well-defined population above the CCS is required for the persistence of measles, invasion into such a population would almost inevitably result in the extinction of the disease due to the exhaustion of susceptibles. Using a simple deterministic model, we have shown that high birth and transmission rates would maximize the probability of a successful colonization by minimizing the time until a second epidemic could take off.

There is very little evidence as to the biological mechanism that could restart the second invasion. However, the analysis presented here defines the time-scale over which it must have operated. This argument alone is enough to argue against host–parasite evolution or multiple independent zoonotic events, which, based on the modern life history of the virus, operate on far longer time-scales. We would argue that the best explanation for measles lies in the hierarchy of spatial scales found in real populations (McCormack Adams 1981; Watts *et al*. 2005). The development of agriculture, which brought animal species and their pathogens in close proximity to human populations, also brought the development of cities. Imposing a spatial heterogeneity on populations that could slow the rate of spread of disease in the invasion phase could reduce or even eliminate the exhaustion of susceptibles which drives local extinction.

The strong immunization following infection would have been the greatest barrier to measles invasion and more generally for any highly transmissible acute pathogen. The more efficiently they spread, the faster they exhaust the fuel that sustains them. The dynamics underpinning the transition from invasion to endemic persistence for immunizing emerging infections is still comparatively unexplored. In this paper, we have demonstrated that for highly infectious diseases to jump between different host species, the right combination of deterministic invasion and stochastic persistence thresholds must be satisfied.

## Figures and Tables

**Figure 1:**
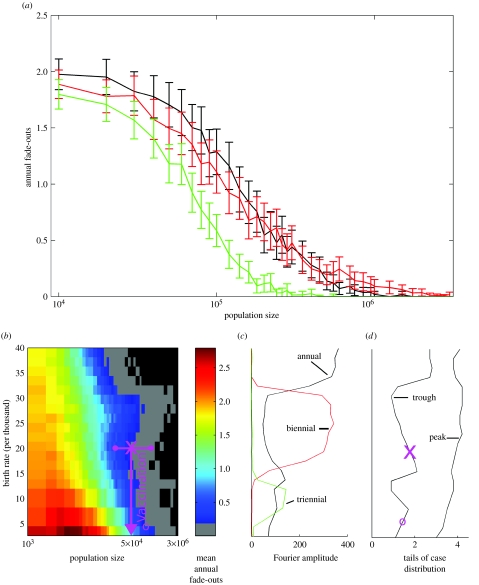
Changing patterns of persistence with birth rate. (*a*) Annual fade-outs against population size: calculated from 50 replicates of a 24-year time-series of the stochastic SEIR model for three representative birth rates. Black, red and green curves correspond to a birth rate of 20, 30 and 40 per thousand, respectively. Error bars indicate standard errors (calculated using the variance between the replicates). (*b*) Mean annual fade-outs for population size against birth rate (50 replicates, 24-year time-series). The England and Wales CCS (500 000) is marked with a cross, with bands of uncertainty due to clumping of the population sizes in the data. Black shades represent no fade-outs over 50 replicates. Grey shades represent up to 0.3 annual fade-outs in 50 replicates (below which fade-out would be unlikely in a short time-series). The expected CCS for England and Wales after vaccination is indicated by a circle. (*c*) The average power of the largest population size (3 million) around the annual (black), biennial (red) and triennial (green) modes of the Fourier spectrum against birth rate. (*d*) Trough and peak size estimated from case distribution, plotted against birth rate. The trough size at the England and Wales fit (cross) and after vaccination (circle) at 70% are indicated.

**Figure 2:**
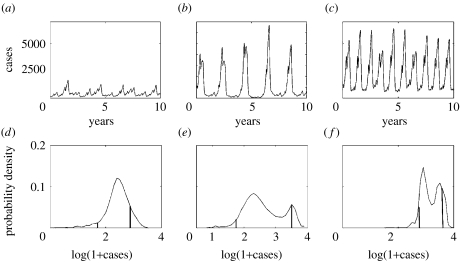
Annual, biennial and triennial dynamics and the definition of the trough and peak size. (*a*–*c*) Ten-year time-series of case reports from one replicate of the stochastic model illustrating the triennial, biennial and annual epidemics experienced as the birth rate is increased (*N*=3 million, *B*=8, 20 and 42 per thousand). (*d–f*) The corresponding case distributions for triennial, biennial and annual epidemics. Averaged over 50 replicates of the stochastic model, this distribution gives the probability of a given value of *x*(*t*)=log(1+cases(*t*)) occurring in a given week. The tails of the distribution used to define the peak and trough size are indicated.

**Figure 3:**
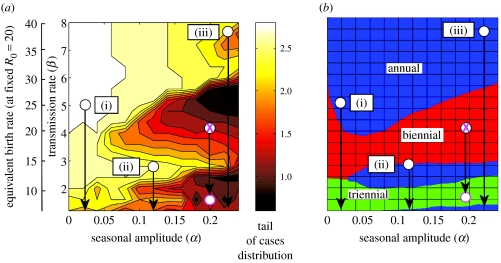
Expected dynamical transitions and implications for the impact of vaccination on persistence from high birth rate countries. (*a*) Trough depth (tail of case distribution) for a large population *N*=3 million at a fixed birth rate (20 per thousand) for a range of transmission rates (*β*≈*R*_0_/5.0 contacts per day) and seasonal amplitudes (*α*). The equivalent range of birth rates for a fixed *R*_0_=20 is given on the second right-hand axis (per thousand population). (*b*) Complementing (*a*), we plot the dominant mode of the Fourier spectrum against transmission rate (*β*) and seasonal amplitude of variation (*α*). Blue, predominantly annual epidemics; red, biennial; green, triennial. The three topologically different paths following vaccination from a community experiencing annual dynamics at high birth rate (*b*=40 per thousand) are indicated. (i) *R*_0_≈12, (ii) *R*_0_≈6 and (iii) *R*_0_≈20. The path corresponding to the predicted impact of vaccination on England and Wales (*b*=20 per thousand, *R*_0_=20) is also marked as cross to circle.

**Figure 4:**
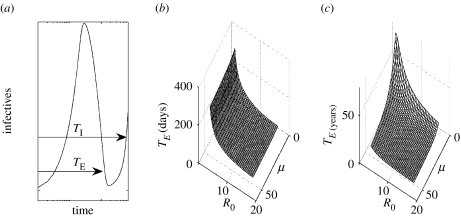
Persistence during invasion: interplay of transmission. and birth rates. (*a*) The first invasion of a well-mixed population results in inevitable extinction due to exhaustion of susceptibles after a time *T*_*E*_ and first possible re-invasion a time *T*_*I*_ later. (*b*) The dependence of *T*_*E*_, the time to the first extinction, on transmission and birth rates, defined as the first time when (*I*≤1, *E*≤1). (*c*) The dependence of *T*_*I*_, the time to first possible re-invasion, on transmission and birth rates, defined as the first time after *T*_*E*_ that (d*I*/d*t*)>0

## Supplementary Material

Technical appendix Seasonality and the persistence and invasion of measles
